# Catamenial rectal bleeding due to invasive endometriosis: a case report

**DOI:** 10.1186/s13256-020-02386-w

**Published:** 2020-05-26

**Authors:** Joshua J. Keith, Lorenzo O. Hernandez, Livia Y. Maruoka Nishi, Tarang P. Jethwa, Jason T. Lewis, George G. A. Pujalte

**Affiliations:** 1 Department of Family Medicine, Mayo Clinic, 4500 San Pablo Road, Jacksonville, Florida 32224 USA; 2 BayCare, Allendale Primary Care, Saint Petersburg, Florida USA; 3 Department of Laboratory Medicine and Pathology, Mayo Clinic, Jacksonville, Florida USA

**Keywords:** Cyclic rectal bleeding, Deep infiltrative endometriosis, Bowel endometriosis, Invasive endometriosis, Endometrial implants, Case report

## Abstract

**Background:**

Although gastrointestinal involvement is the most common site for extra-genital endometriosis, deep infiltrative endometriosis, which affects the mucosal layer, is very rare.

**Case presentation:**

We present a case of a 41-year-old white woman with cyclic rectal bleeding. Magnetic resonance imaging was done, together with colonoscopy and histologic staining of biopsied samples, which led to the final diagnosis of intestinal invasive endometriosis with recto-sigmoid stricture. Our patient was treated symptomatically with stool softeners.

**Conclusion:**

This case provides a rare example of catamenial bleeding. It is important to keep invasive endometriosis on the differential diagnosis whenever a premenopausal woman has cyclical rectal bleeding.

## Introduction

Endometriosis is defined as the presence of functional endometrial glands and stroma outside the uterine cavity [1]. Although gastrointestinal involvement is the most common site for extra-genital endometriosis, deep infiltrative endometriosis (DIE), which affects the mucosal layer, is very rare [2]. Bowel endometriosis can lead to significant complications, including gastrointestinal bleeding, bowel obstruction, perforation, and/or malignant transformation [3]. The lack of pathognomonic manifestations in intestinal endometriosis makes the differential diagnosis from other diseases challenging [4].

In this report we present a case of a middle-aged woman with rectal endometriosis after having undergone a total hysterectomy with bilateral salpingectomy and left oophorectomy 1 year prior.

## Case presentation

A 41-year-old white woman with a past medical history of endometriosis presented to our clinic for her annual examination. She wanted to discuss cyclic rectal bleeding after having undergone a total hysterectomy with bilateral salpingectomy and left oophorectomy 1 year prior. She stated that, over the past 6–8 months, she had been experiencing bloody bowel movements for 1 week each month, in the same pattern as her previous menstrual cycles. She also experienced sharp, lower abdominal pain with these bloody bowel movements, similar to the pain from her endometriosis in the past. She described a mild to moderate amount of bleeding and noted that the blood was typically mixed with stool. The blood was dark red, which she believed looked very similar to her menses. She reported normal bowel movements the other 3 weeks of the month. Prior to her hysterectomy, she completed a colonoscopy which showed no transmural implants. Previous treatment for endometriosis included oral contraceptives which gave no significant symptomatic relief. Her other past medical history included hypertension for which she was taking extended-release metoprolol 50 mg in the morning and 25 mg in the evening before bed. She had never smoked tobacco and consumed alcohol occasionally. At that time, she worked as a systems engineer for information technology.

She had the onset of menarche at age 10 with heavy periods until age 16, at which time she went on oral contraceptive pills. At age 37, she gave birth to twins at 29 weeks of gestation via cesarean section without complications. Her other surgical history included a tonsillectomy at age 3, cervical conization at age 22, rhinoplasty at age 26, exploratory laparoscopy for excision of stage IV endometriosis with en bloc excision, left ovarian cystectomy, and bilateral ovarian suspension at age 34, as well as total hysterectomy, as mentioned above.

She denied any family history of endometriosis, although she noted that her mother had heavy periods prior to giving birth to our patient. Her mother also suffered from asthma. Her father had heart disease and her grandparents had a history of heart disease, diabetes, stroke, high cholesterol, hypertension, osteoporosis, and alcohol abuse.

Our patient’s examination revealed a temperature of 36.7 ºC, heart rate of 57 beats per minute (bpm), and blood pressure of 129/78 mmHg. She was alert and oriented with no focal neurologic deficits. Cardiac and lung examinations were normal. An abdominal examination revealed normoactive bowel sounds with no tenderness to palpation. No external hemorrhoids were visualized on rectal examination and stool guaiac was negative. An anoscopy was not performed.

Our differential diagnoses when we first saw her were: invasive endometriosis, internal hemorrhoids, diverticulosis, adenocarcinoma of the colon, inflammatory bowel disease, and angiodysplasia.

**Fig. 1:**
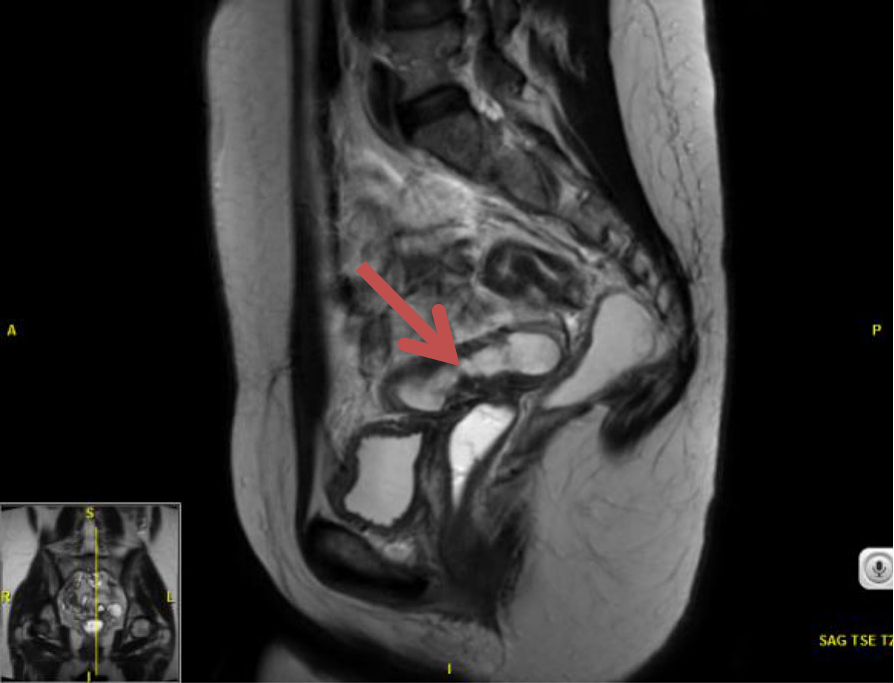
Sagittal T2-weighted magnetic resonance imaging section of pelvis with recto-sigmoid colon endometrial implant (*red arrow*)

**Fig. 2:**
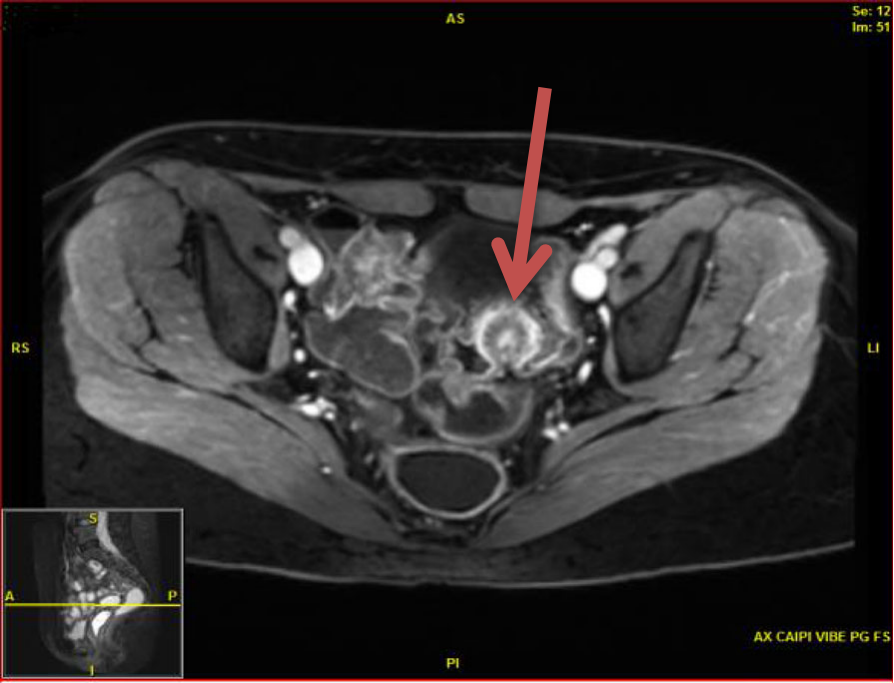
Transverse T2-weighted magnetic resonance imaging section of pelvis with recto-sigmoid colon endometriosis (*red arrow*)

**Fig. 3:**
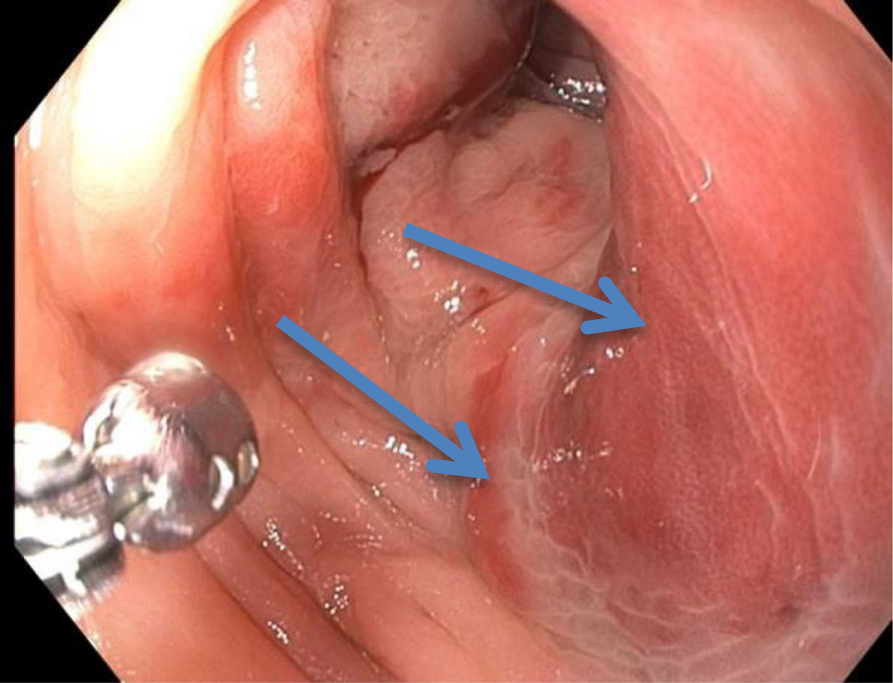
Sigmoid colon on colonoscopy showing erythematous changes (*blue arrows*)

**Fig. 4:**
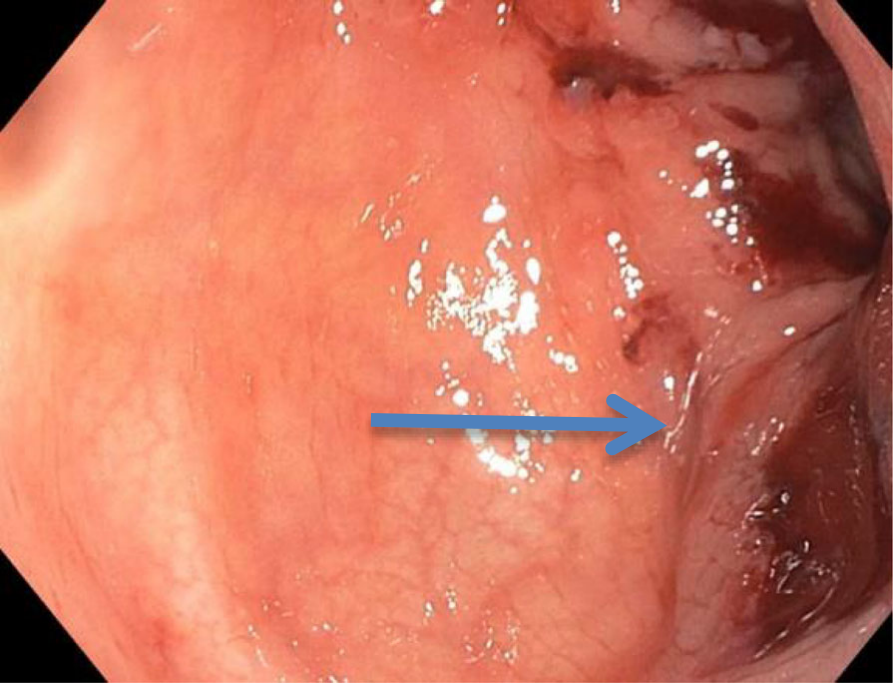
Sigmoid colon on colonoscopy showing erythematous changes (*blue arrow*)

**Fig. 5:**
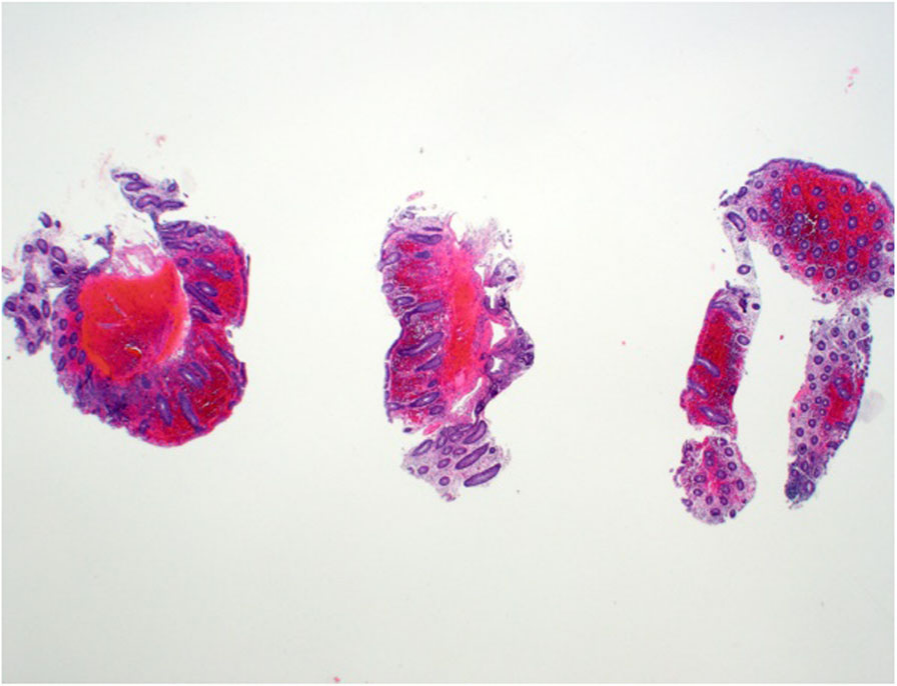
Low power hematoxylin and eosin view. Relatively diffuse permeation of multiple colonic fragments of hemorrhage

**Fig. 6:**
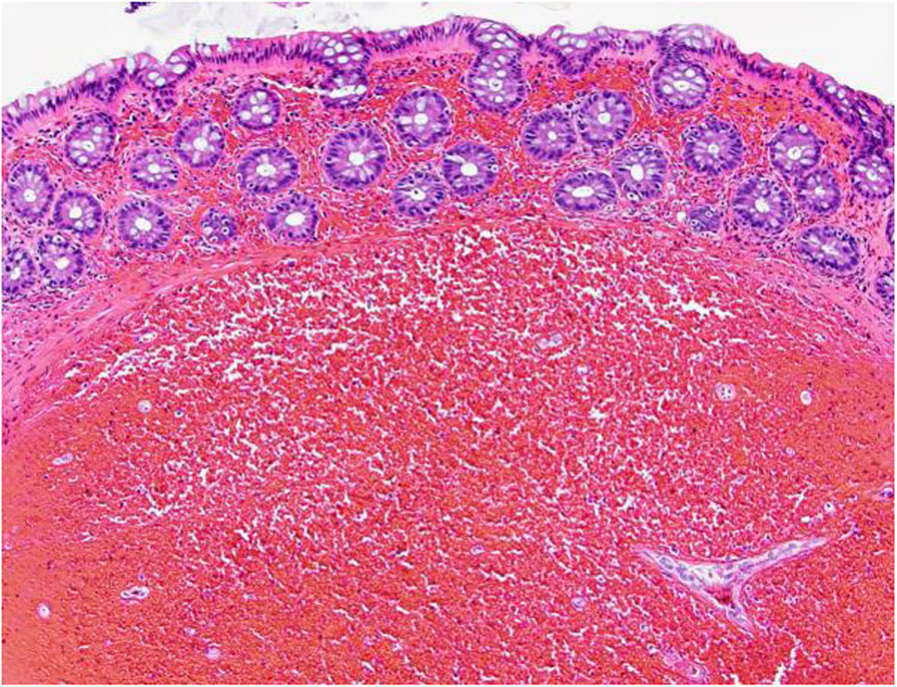
High power hematoxylin and eosin view. The submucosa (lower two-thirds of image) is expanded with blood, which has also filled the lamina propria of the overlying mucosa

Laboratory tests revealed a largely normal complete blood count (CBC) with a hemoglobin of 12.4, platelet count was 196,000, and white blood cell count was 10,000. Our patient’s electrolytes and kidney function were normal with creatinine of 0.9, blood urea nitrogen (BUN) of 13, and albumin of 4.1. Other examinations such as urine analysis, serology, and microbiology were not drawn. Magnetic resonance imaging (MRI) of her pelvis, with and without contrast, was performed. Findings were consistent with invasive endometriosis in the pelvis, with possible sigmoid colon invasion (Figs. 1 and 2). A colonoscopy was performed and revealed a stricture in the recto-sigmoid colon and endometrial implants (Figs. 3 and 4). This was thought likely to be extrinsic infiltrating endometriosis affecting the submucosal and mucosal layers with erythematous mucosal changes. These sites were biopsied. The final pathology report revealed fragments of colonic mucosa with marked lamina propria and submucosal congestion (Figs. 5 and 6). Immunostaining for estrogen receptor was negative.

Our patient was referred for follow-up with both Gynecology and Colorectal Surgery. She was advised by the surgical team to undergo exploratory laparotomy with removal of any endometrial implants and resection of the affected colon to avoid worsening stricture and potential obstruction. They recommended sparing of the remaining ovary to avoid surgical castration which, in women under the age of 45, has been shown to correlate with increased all-cause mortality [5, 6].

She opted to not proceed with the surgery due to concern of recurrence by maintaining her right ovary; she decided to manage the bleeding with stool softeners, increasing the dosage during her menstrual periods. After deciding for the non-surgical approach, our patient never complained about the same symptoms again, suggesting that the stool softeners managed her symptoms. Since her diagnosis over 4 years ago, her pain and bleeding have improved and she is starting to feel the first symptoms of menopause including hot flashes, mood swings, and difficulty sleeping. Since her menopausal symptoms have been mild and intermittent, she has decided to hold off from seeking treatment for them at this time.

## Discussion

Our patient was a 41-year-old woman with a history of endometriosis status post hysterectomy, bilateral salpingectomy, and unilateral oophorectomy. Approximately 9–11 months after surgery, she developed lower abdominal pain and rectal bleeding. Colonoscopy revealed a stricture in the recto-sigmoid colon and endometrial implants. She elected to manage her symptoms non-surgically. This particular case is unusual due to the rare presentation of deeply infiltrative lesions causing rectal bleeding with visible endometrial mucosal implants on colonoscopy [2]. It also illustrates the importance of recognizing that patients who have had a unilateral oophorectomy of the affected ovary remain at risk for recurrent endometriosis. The prevalence of endometriosis is 10% in reproductive-aged women, and the most common site of extra-genital endometriosis is the gastrointestinal tract, which is found in 3.8%–37% of patients with endometriosis [2]. The prevalence of deep endometriosis is estimated to be around 1–2% [7]. The symptoms of bowel endometriosis can vary depending on the site and can include anal pain, low back pain, lower abdominal pain, bleeding per rectum, and dyspareunia, occurring mainly during menstruation [3]. However, the cyclical pain is not pathognomonic of endometriosis, appearing also within inflammatory bowel disease and irritable bowel syndrome that can worsen during menses [4]. According to Slack *et al.* (2007), rectovaginal endometriosis presents in 5–10% of women with endometriosis and has more severe symptoms than the superficial form of disease with increased risks of bowel and urinary tract involvement [8].

Imaging studies performed to diagnose endometriosis may include a computed tomography (CT) scan, MRI, a transvaginal ultrasound, and/or an endorectal ultrasound. MRI has a high sensitivity (80%) and high specificity (90%) for detecting pelvic endometriosis [3]. However, a Cochrane review from 2016 established that no imaging tests were superior to surgery in the diagnosis of endometriosis [9].

Colonoscopy can also be helpful if imaging confirms lesions in the intestinal tract. It is important to note that endoscopically obtained biopsy material is superficial and endometriosis usually involves the deeper layers of the bowel wall [3, 10, 11, 12]. Tissue obtained by endoscopy often reflects chronic injury but may lack diagnostic endometriotic foci which could introduce the potential for misinterpretation and misdiagnosis [6, 12, 13–16]. In addition, the endometriotic implants can promote secondary mucosal changes, which may resemble differential diagnoses, such as ischemic colitis, inflammatory bowel disease, or even neoplasm [4, 12, 16].

Treatment of endometriosis is dependent on the severity of the disease. Studies show that patient quality of life and satisfaction rates are comparable with medical and surgical treatment [17]. Medication treatment offers success in early uncomplicated cases and includes oral contraceptive pills, progestins, danazol, and gonadotropin-releasing hormone (GnRH) agonists. Surgery is the preferred treatment when hormonal therapy fails. Bowel resection is considered to be the recommended therapy in patients with bleeding, bowel obstruction, and suspicion of malignancy [3, 11].

This patient was diagnosed as having intestinal endometriosis with mucosal involvement. This case is particularly relevant because she underwent a hysterectomy for endometriosis 7 months before the symptoms of cyclic bowel pain and bleeding began. In addition, her symptoms together with the recto-sigmoid stricture may suggest other diagnoses that must be ruled out, such as colon cancer and irritable bowel syndrome [15, 16]. Sometimes, if the provider is not used to dealing with endometriosis cases, this diagnosis may be left out. Moreover, DIE is rare and can be a diagnostic challenge, making this report potentially useful as an example of how invasive bowel endometriosis might be approached and managed in clinic.

## Conclusion

Catamenial bleeding secondary to invasive endometriosis is a rare cause of rectal bleeding. It is important to keep invasive endometriosis on the differential diagnosis whenever a premenopausal woman has cyclical bleeding.

## Data Availability

Not applicable.
